# TiO_2_ ALD Coating of Amorphous TiO_2_ Nanotube Layers: Inhibition of the Structural and Morphological Changes Due to Water Annealing

**DOI:** 10.3389/fchem.2019.00038

**Published:** 2019-02-01

**Authors:** Siowwoon Ng, Hanna Sopha, Raul Zazpe, Zdenek Spotz, Vijay Bijalwan, Filip Dvorak, Ludek Hromadko, Jan Prikryl, Jan M. Macak

**Affiliations:** ^1^Central European Institute of Technology, Brno University of Technology, Brno, Czechia; ^2^Faculty of Chemical Technology, Center of Materials and Nanotechnologies, University of Pardubice, Pardubice, Czechia

**Keywords:** TiO_2_, nanotubes, atomic layer deposition, coating, water annealing

## Abstract

The present work presents a strategy to stabilize amorphous anodic self-organized TiO_2_ nanotube layers against morphological changes and crystallization upon extensive water soaking. The growth of needle-like nanoparticles was observed on the outer and inner walls of amorphous nanotube layers after extensive water soakings, in line with the literature on water annealing. In contrary, when TiO_2_ nanotube layers uniformly coated by thin TiO_2_ using atomic layer deposition (ALD) were soaked in water, the growth rates of needle-like nanoparticles were substantially reduced. We investigated the soaking effects of ALD TiO_2_ coatings with different thicknesses and deposition temperatures. Sufficiently thick TiO_2_ coatings (≈8.4 nm) deposited at different ALD process temperatures efficiently hamper the reactions between water and F^−^ ions, maintain the amorphous state, and preserve the original tubular morphology. This work demonstrates the possibility of having robust amorphous 1D TiO_2_ nanotube layers that are very stable in water. This is very practical for diverse biomedical applications that are accompanied by extensive contact with an aqueous environment.

## Introduction

Various morphologies of TiO_2_ with nano scale dimensions have been extensively investigated as photo catalysts for H_2_ evolution, dye-sensitized solar cells (DSSCs), degradation of organic compounds, methanol oxidation, CO_2_ reduction, self-cleaning and anti-fogging, and many other applications (Chen and Mao, 2007; Schneider et al., 2014; Wang et al., 2014). Particularly in the last 15 years, the anodic self-organized TiO_2_ nanotube layers have attracted scientific interests in the mentioned areas. This is mainly attributed to the controllable geometry and large specific surface area of the anodic TiO_2_ nanotube layers which allow higher reaction activities as well as the one-dimensional (1D) orientation which offers unidirectional charge transport from the tubes to the supporting Ti substrate (Macak et al., 2007; Lee et al., 2014; Wang et al., 2014).

Generally, the as-anodized TiO_2_ nanotube layers in the amorphous state are not favored in semiconducting applications such as photo catalysts and DSSCs, primarily due to their low conductivity and a significant number of recombination centers which impede efficient charge transport (Roy et al., 2011; Krbal et al., 2016). As the electronic properties are influenced by the structural quality of the nanotube layers (Tsuchiya et al., 2007), post-thermal annealing in temperature range of 280–600°C for 1–3 h (Varghese et al., 2003; Tighineanu et al., 2010) or hydrothermal treatment (Yu et al., 2010) needs to be carried out to crystallize the nanotube layers.

For a long time, only crystalline TiO_2_ nanomaterials have been comprehensively studied, whereas its amorphous counterparts have not received much attention so far. In spite of the strong focus on crystalline TiO_2_ forms (anatine or rutile) that show higher performance in diverse applications, amorphous TiO_2_ structures have increasingly showcased its popular role in various semiconductor applications as well (Lu et al., 2008; Ortiz et al., 2008; Djenizian et al., 2011; Xiong et al., 2011; Bi et al., 2013; Wang et al., 2015; Jiang et al., 2016; Liang et al., 2018; Liu et al., 2018). TiO_2_ has been long recognized as excellent biocompatible material owing to its low cytotoxicity, high stability, and antibacterial properties (Fu and Mo, 2018). Its amorphous state is particularly preferred in biomedical applications, including carrier for magnetic nanoparticles for protein purification (Kupcik et al., 2017), supporting layer for enhanced hydroxyapatite (Hap) deposition in Osseo integration (Kar et al., 2006; Crawford et al., 2007), supporting layer for epithelial cells and fibroblasts viability (Mei et al., 2014) and improved magnetic resonance contrast for molecular receptor targeted imaging (Chandran et al., 2011). In the case of TiO_2_ nanotube layers, controllable nano-geometry, surface modification, topography, and roughness are crucial for tissue and cell vitality (seeding, spreading, and proliferation) (Park et al., 2007; Peng et al., 2009; Fu and Mo, 2018). On top of its hemocompatibility (Huang et al., 2017), the tubular morphology is an added advantage for genes, drugs, and therapeutic carrier or reservoirs, for example, gentamicin sulfate, chitosan, bone morphogenetic protein 2, and tumor necrosis factor-related apoptosis-inducing ligand (Hu et al., 2012; Feng et al., 2016; Kaur et al., 2016).

For the mentioned biomedical applications, the TiO_2_ nanotube layers are frequently used in an aqueous environment. Nevertheless, the soaking of the as-anodized amorphous TiO_2_ nanotube layers in a water bath transform them to polycrystalline anatase structure via so-called water annealing effect or low-temperature crystallization approach (Liao et al., 2011; Wang et al., 2011; Krengvirat et al., 2013; Lamberti et al., 2015; Cao et al., 2016). These water annealing processes are accompanied by a strong morphological transformation. As a result, the unique tubular morphology cannot be sustained in the case of prolonged soaking. Additional particle-like deposits grow on the surface of the amorphous nanotubes and may completely block them, reducing drastically the accessibility of various species inside the nanotubes and reducing also the overal available surface area. Eventually, in certain cases, the amorphous nanotubes were first transformed to double-wall nanotubes, then to core-shell wires/rods-within-tubes and finally full transformation into crystallized nanowires/rods after different soaking durations took place (Wang et al., 2011; Lamberti et al., 2015; Cao et al., 2016). The water annealed nanotubes or nanowires/rods possess much rougher surface as compared to the amorphous nanotube layers. Interestingly, when the similar soaking experiment was carried out in a cell culture environment, such as in fetal bovine serum and phosphate buffered saline (PBS) media, the amorphous TiO_2_ nanotube layers did not experience any structural or morphological changes (Cao et al., 2016).

To incorporate the aforementioned advantages of anodic 1D TiO_2_ nanotube layers in the biomedical applications, it is crucial to maintain the amorphous state and preserve the tubular morphology. In fact, it is quite common that the addition of a shell (an outer layer) serves as a protective layer for the inner core structure (Yan et al., 2012; Hu et al., 2014; Kwiatkowski et al., 2015). For instance, an ultrathin Al_2_O_3_ coating was employed to improve the chemical, mechanical, and thermal stability of TiO_2_ nanotube layers in extreme environments (Zazpe et al., 2017) such as for Li-ion batteries (Sopha et al., 2017a). On the other hand, a SiO_2_ insulating layer was utilized to encapsulate TiO_2_ nanoparticles to inhibit the photo catalytic activity, which undesirably darkens the white pigment of TiO_2_ (Guo et al., 2017), and also to improve the cell compatibility and photo-killing ability (Feng et al., 2013).

To achieve ultrathin and continuous coatings that completely enfold a high aspect ratio structure such as TiO_2_ nanotube layers, ALD technique is viable to provide such homogeneous and conformal coatings due to its self-saturating surface reactions (Leskelä and Ritala, 2002; Leskelä et al., 2007; Zazpe et al., 2016, 2018). Tupala et al. first performed an ALD amorphous TiO_2_ coating within crystalline TiO_2_ nanotube layers. It is worth noting that with a 5 nm amorphous TiO_2_ layer, the conductivity of the coated nanotube layer is substantially increased. (Tupala et al., 2012) We have also demonstrated that additional ALD crystalline TiO_2_ coatings within crystalline TiO_2_ nanotube layers passivate defects within TiO_2_ and enhance the charge carrier separation towards improved photo electrochemical and photo catalytic performance (Sopha et al., 2017b).

Despite other materials such as ZnO, Fe_3_O_4_, and CuO may potentially serve as a protective coating, we deliberately select an identical coating material (TiO_2_) for the core nanotube layers due to the fact that (i) the biocompatible TiO_2_ coating is required to be robust in extreme environments, and (ii) the stacking of two different materials (different densities) creates a gradient at the interface between outer and inner layers, which complicates the reactants transfer and interaction process. In the present work, we extend the application of ALD TiO_2_ coatings as a protective coating of amorphous TiO_2_ nanotube layers to prevent their morphological changes, known as water annealing effect. The longest soaking duration shown in previous works was up to 7 days (Wang et al., 2011; Cao et al., 2016). We significantly prolong the soaking duration up to 28 days to show the extreme stability of these ultrathin TiO_2_ coated TiO_2_ nanotube layers in order to broaden their functional range specifically in the aqueous environments for biomedical applications.

## Materials and Methods

Self-organized TiO_2_ nanotube layers with thicknesses of ≈5 μm and inner diameters ≈230 nm were produced via electrochemical anodization as described in our previous works (Das et al., 2017). Atomic layer deposition (ALD, TFS200, Benes) of TiO_2_ was carried out at 150°, 225°, and 300°C using TiCl_4_ (electronic grade 99.9998%, STREM) and Millipore deionized water (15 MΩ) as the titanium precursor and the oxygen source, respectively. Temperature of both precursors was kept at 20°C. High purity N_2_ (99.9999%) was the carrier and purging gas at a flow rate of 400 standard cubic centimeters per minute sccm (Standard Cubic Centimeters per Minute). Under these deposition conditions, one ALD growth cycle was defined by the following sequence: TiCl_4_ pulse (500 mS)-N_2_ purge (3 s)-H_2_O pulse (500 mS)-N_2_ purge (4 s). The as-anodized amorphous TiO_2_ nanotube layers were coated by TiO_2_ applying different ALD cycles, N_ALD_ = 10, 50, and 150, yielding nominal thicknesses of 0.56, 2.8, and 8.4 nm, respectively. The thickness is obtained according to the growth rate per ALD cycle, evaluated from TiO_2_ thin layers deposited on Si wafers using variable angle spectroscopic ellipsometry using VASE® ellipsometer, J.A. Woollam.

For water soaking, the blank and TiO_2_ coated TiO_2_ nanotube layers were soaked in deionized water (18 MΩ.cm) and phosphate buffered saline (PBS) for different durations, i.e., 1, 7, 14, or 28 day(s) in a still environment at room temperature. The morphology of the blank, coated, and soaked TiO_2_ nanotube layers was imaged by field-emission scanning electron microscope (SEM, JEOL JSM 7500F, FEI Verios 460L). The structural evaluation was based on X-ray diffraction (XRD) measured by diffractometer (SmartLab 3kW from Rigaku). The diffractometer was set up in Bragg-Brentano geometry using Cu Kα radiation (λ = 1.54 Å) equipped by 1D-detector Dtex-Ultra. Cu lamp was operated at current 30 mA and voltage 40 kV. Phase analysis was performed based on chemical composition using databases PDF2 and ICSD. The chemical state of the blank and TiO_2_ coated (N_ALD_ = 150 at 300°C) amorphous nanotube layers was examined by X-ray photoelectron spectroscopy (XPS, ESCA2SR, Scienta-Omicron) using a monochromatic Al Kα (1486.7 eV) X-ray source. The survey spectra were acquired using 250 W power of X-ray source with pass energy set to 150 eV. The quantitative analysis was based on sensitivity factors provided by the manufacturer. It is noteworthy to point out that the quantitative analysis was performed in order to provide a relative comparison between a chemical composition of blank and TiO_2_ coated nanotube layers. The absolute values of the atomic concentration of elements are in great extent affected by the surface sensitivity of XPS.

## Results and Discussion

**Figure 1:**
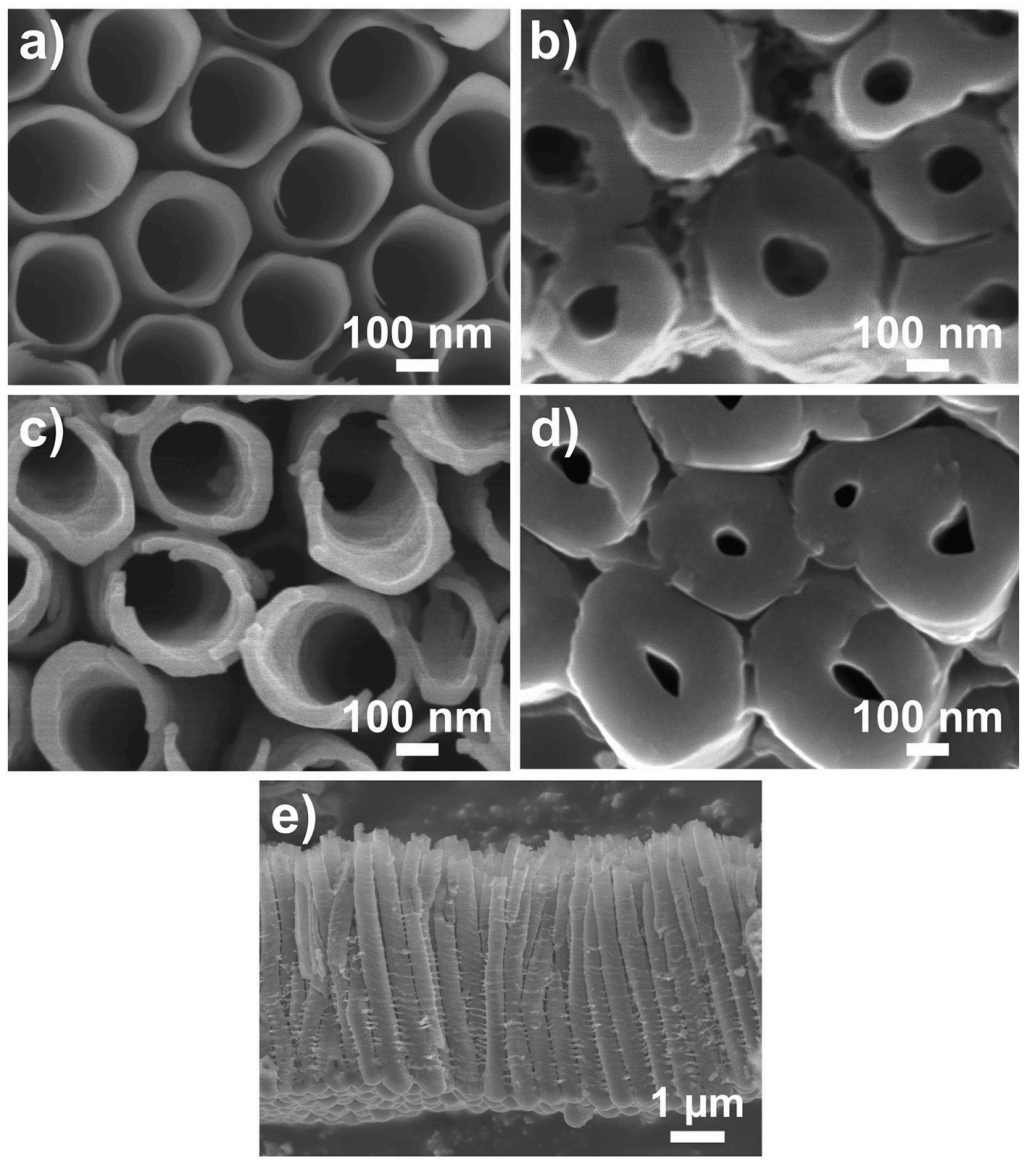
SEM images of **(a,b)** blank and **(c,d)** TiO_2_ coated (N_ALD_ = 150 ≈8.4 nm) TiO_2_ nanotube layers. **(a,c)** Are taken at top of the nanotube layer (water/nanotubes opening interface) **(b,d)** Are taken at the bottom of the nanotube layer (bottom of nanotubes/Ti interface). **(e)** Shows the entire nanotube layer with thickness ≈5 μm.

The blank (as-anodized) TiO_2_ nanotube layers and TiO_2_ coated (N_ALD_ = 150 at 300°C) TiO_2_ nanotube layers are imaged by SEM in two regions of the nanotube layer, i.e., the top (water/nanotubes opening interface, Figures 1a,c) and the bottom (bottom of nanotubes/Ti interface, Figures 1b,d). At the top of the tubes, the blank nanotube layer clearly presents an inner diameter of ≈230 nm. It is obvious that the ALD TiO_2_ (≈8.4 nm) coated nanotube layer shows slightly thicker tube walls compared to the blank nanotube layer. In addition, at the bottom of the tubes, the ALD coated nanotube layer has a smaller inner diameter. These images evidence that the coating is uniform across the entire tube walls. The layer thicknesses are ≈5 μm as shown in Figure 1e. The coating thickness is confirmed by the thickness measurement on the identical TiO_2_ coating deposited on a flat substrate. It is an utmost challenge to differentiate the TiO_2_ coating and TiO_2_ tube wall due to the identical material, as they are of the same mass and contrast. This fact disables microscopists to distinguish them. Nevertheless, in a previous work, it was shown that the walls of 400 ALD cycles (≈22 nm) TiO_2_ coated TiO_2_ nanotube layer is visibly much thicker than the blank nanotube layer (Sopha et al., 2017b). Thus, from the ALD principle, the thickness of the present coatings follows the same trend: the higher number of ALD cycles, the thicker is the coating.

**Figure 2:**
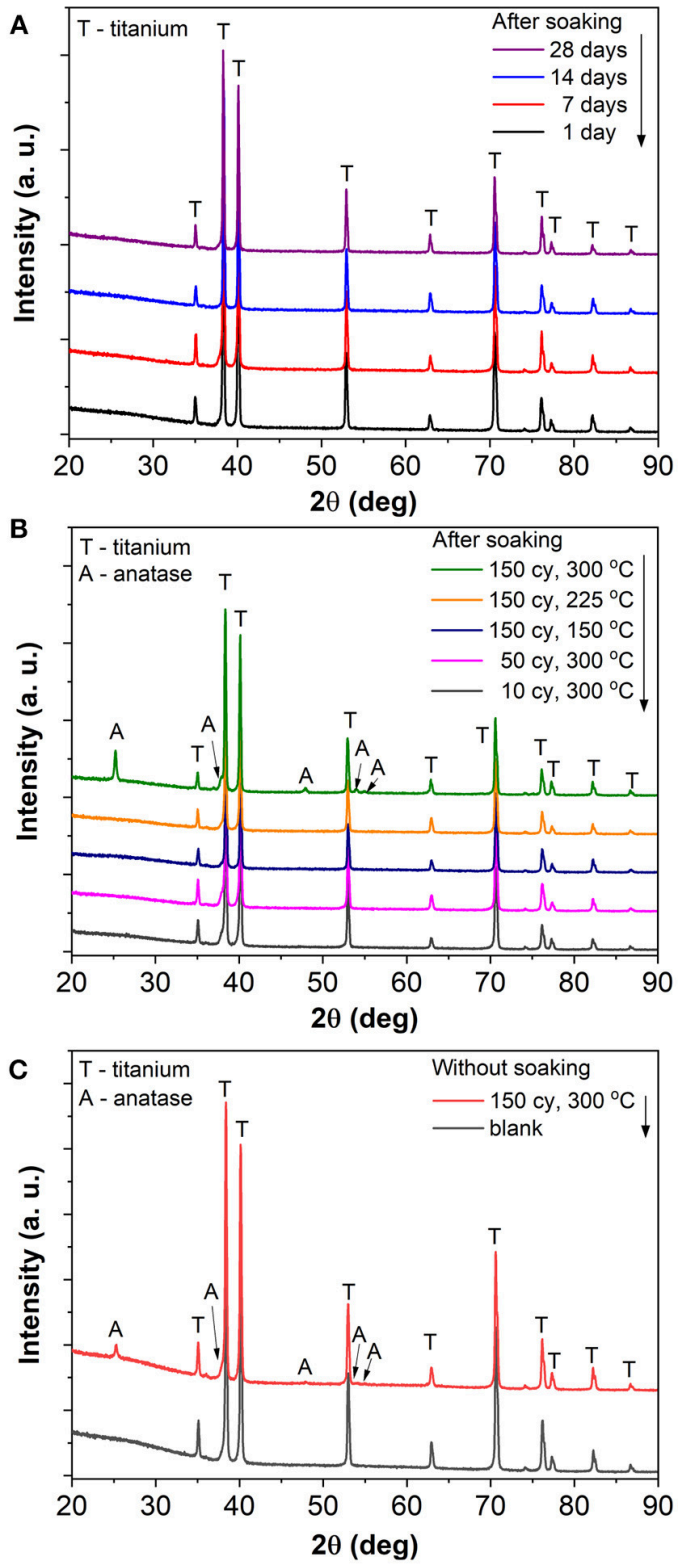
XRD patterns of **(A)** blank TiO_2_ nanotube layer soaked for 1, 7, 14, and 28 day(s), **(B)** TiO_2_ coated TiO_2_ nanotube layers with different coating thicknesses (N_ALD_ = 10, 50, 150) and different deposition temperatures (150°, 225°, and 300°C) soaked for 28 days, and **(C)** blank and TiO_2_ coated (N_ALD_ = 150 at 300°C) TiO_2_ nanotube layers (both without any soaking).

The blank and TiO_2_ coated TiO_2_ nanotube layers were then soaked in deionized water for different durations from 1 to 28 days. No modification in physical appearance such as changes in color was noticed. We first proceeded to investigate the structural properties on (i) blank nanotube layers soaked for all durations; (ii) nanotube layers with different ALD coating thickness (N_ALD_ = 10, 50, 150) deposited at 300°C and (iii) nanotube layers with N_ALD_ = 150 deposited at different temperatures (150°, 225°, and 300°C), the latter two cases were soaked for 28 days. The resulting XRD patterns are depicted in Figure 2. The blank nanotube layers remain amorphous after extensive soaking in deionized water up to 28 days, as only the diffraction peaks of hexagonal Ti (from the substrate) are present in the obtained diffraction patterns as shows in Figure 2A. Similarly, all ALD cycles and temperatures coated nanotube layers are in amorphous state after the identical soaking experiments. The only exception is credited to the TiO_2_ coating deposited with N_ALD_ = 150 at 300°C with polycrystalline anatase structure. The corresponding diffraction peaks of anatase are labeled in Figure 2B. Note that the crystallization is not induced by the water soaking; instead, it occurred during the ALD deposition process because the coating with a thickness of 8.4 nm becomes crystalline due to the deposition temperature of 300°C. This is supported by the XRD data of the amorphous nanotube layer, and the same layer then coated with N_ALD_ = 150 at 300°C prior to the soaking experiment, as shown in Figure 2C. The anatase diffraction peaks are very similar before and after soaking. Furthermore, previous studies reported that the initiation of crystallization process is influenced by the ALD deposition temperature and coating thickness (Aarik et al., 2001; Nie et al., 2015). This means that initially an amorphous coating is formed on the substrate until a (thermodynamical) threshold thickness is achieved for the nucleation of crystals. The threshold thickness reduces with the increase of deposition temperature (Aarik et al., 2001; Nie et al., 2015). Certainly, the selection of Ti and O_2_ precursors is another important factor due to the different activation kinetics for different precursors. Several works have suggested that the crystallization process is initiated at the temperature range of 165–250°C (Aarik et al., 1995, 2013; Saha et al., 2014; Chiappim et al., 2016). For example, an 11 nm thin anatase TiO_2_ film was obtained at deposition temperature of 225°C when TiCl_4_ and H_2_O were employed as precursors (Aarik et al., 2013). The whole set of samples was analyzed by XRD, all others were amorphous except for N_ALD_ = 150 at 300°C, and selected patterns are shown in Figure 2. Thus, we can state that all nanotube layers with lower coating thicknesses or at lower deposition temperatures remain amorphous after soaking experiments with the duration up to 28 days.

**Figure 3:**
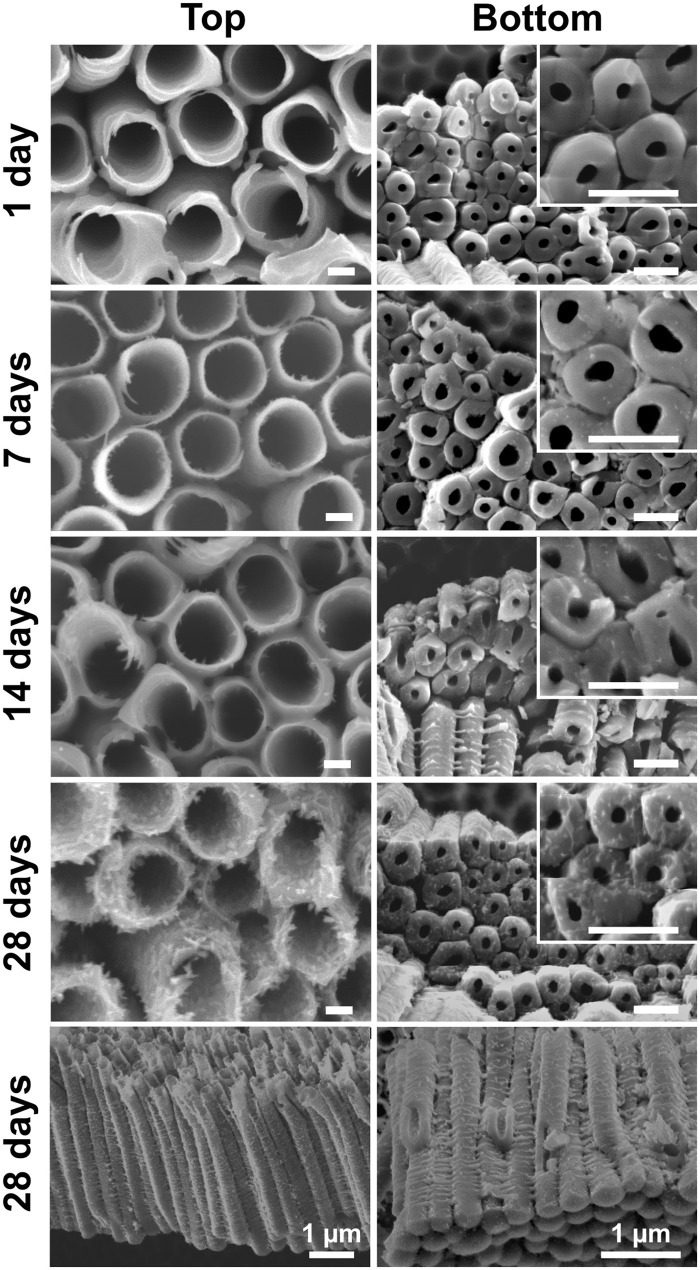
SEM images of blank TiO_2_ nanotube layers soaked in water for 1, 7, 14, and 28 day(s). Scale bars in the left column are 100 nm, in the right column and respective inset are 500 nm, unless stated otherwise.

The soaked blank nanotube layers were further inspected for their morphologies. Figure 3 presents the SEM images of the nanotube layers taken at their top (water/nanotubes opening interface) and bottom (bottom of nanotubes/Ti interface). The larger inner diameter at the top than the bottom featuring a conical shape is a typical type of double-walled nanotube layers (Zazpe et al., 2016). It can be seen that the soaked nanotube layers experience a gradual change. After 1 day of soaking, the blank nanotube layer remains similar to the as-anodized nanotube layer in Figure 1a. When the soaking duration was extended to 7 and 14 days, needle-like particles were observed on the tube walls. A pronounced effect was observed after 28 days of soaking, where the tube walls were completely occupied by the nanoparticles, which is drastically different from the as-anodized nanotube layers. The coalescence of nanoparticles has resulted in rather rough outer and inner tube walls and the nanoparticles were found over the entire nanotubes from the top to the bottom of the tube layers, as visualized in Figure 3 (soakings for 28 days). This confirms that the morphological changes occurred over the entire available nanotube surface that was in contact with the water molecules. And it also confirms very good wettability of these nanotube layers. The final morphology very well-resembles the one reported in the literature (Wang et al., 2011; Liu et al., 2012). The transformation mechanism will be discussed later in this section.

**Figure 4:**
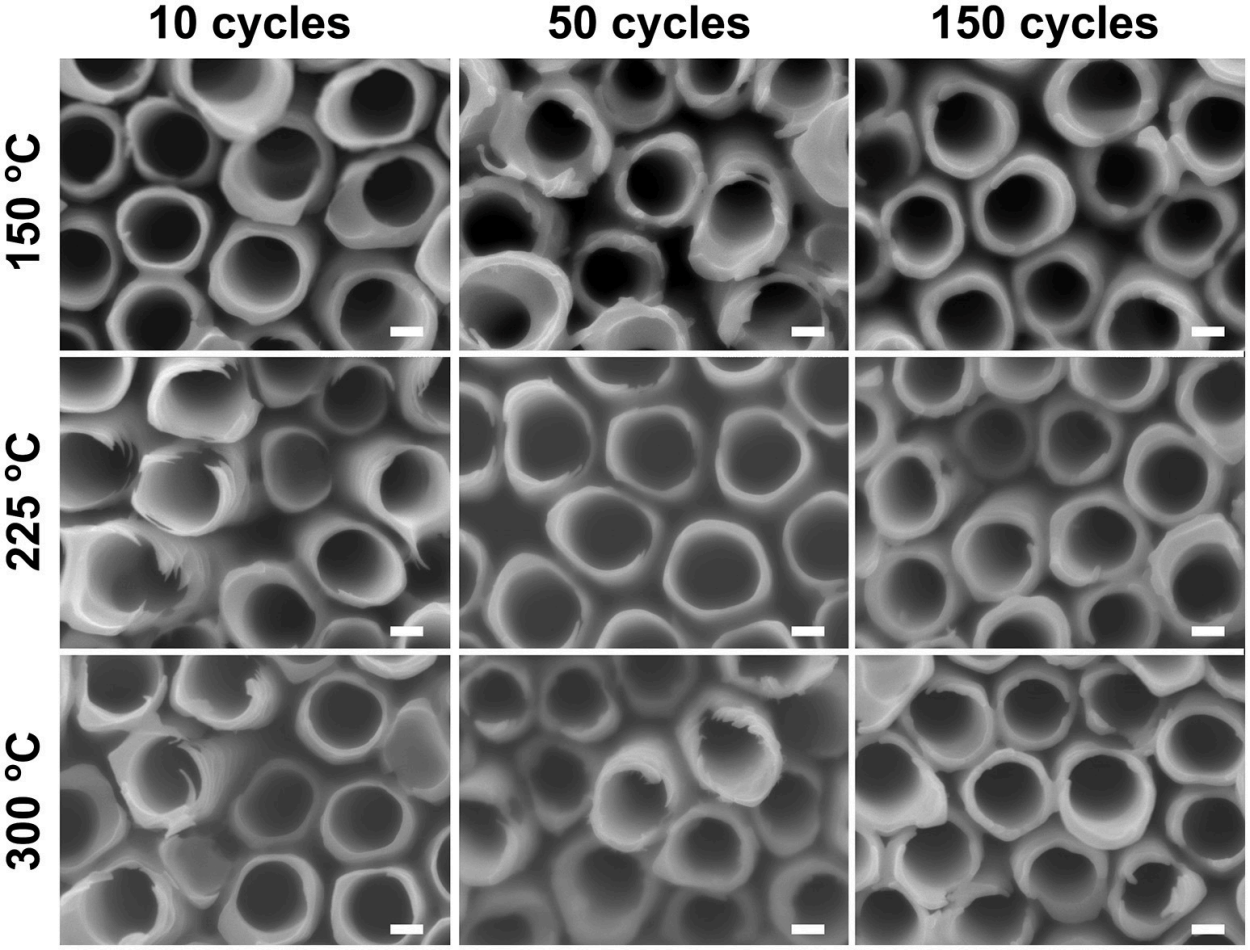
SEM images of atomic layer deposition (ALD) TiO_2_ coated TiO_2_ nanotube layers soaked in water for 14 days, no morphological changes can be observed for these coated TiO_2_ nanotube layers. All scale bars are 100 nm.

Similar water soaking procedures were performed on the TiO_2_ coated TiO_2_ nanotube layers, where the coatings were deposited by ALD at different deposition temperatures and different coating cycles. For these coated nanotube layers, careful inspection did not reveal any noticeable morphological change for soakings up to 14 days, as shown in Figure 4. This implies that the nanotube layers were well-protected by the additional TiO_2_ coatings. Thicker tube walls are observed with the increase of ALD coating cycles (and thickness), but the increase of deposition temperature does not yield any detectable morphological difference in Figure 4.

**Figure 5:**
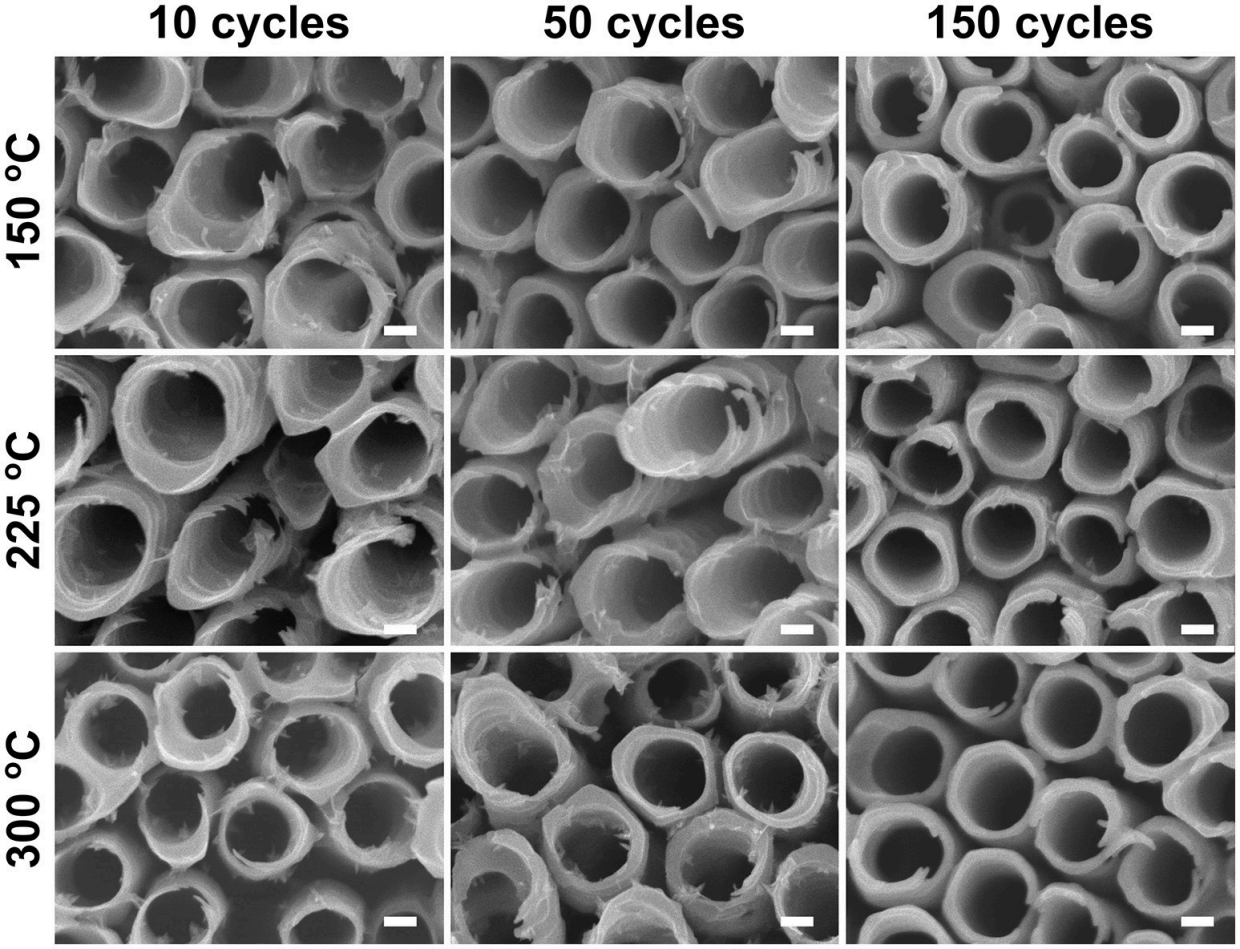
SEM images of ALD TiO_2_ coated TiO_2_ nanotube layers soaked in water for 28 days. All scale bars are 100 nm.

When the soakings were extended to 28 days, a considerable amount of needle-like nanoparticles were grown on the tube walls with low coating thickness (N_ALD_ = 10). The amount of nanoparticles gradually decreased with thicker coatings (N_ALD_ = 50, 150), as shown in Figure 5. However, in comparison to the blank nanotube layers [Figure 3 (28 days)], the amount of needles grown in the coated nanotube layers is significantly lower. The only exception lies in the nanotube layer with TiO_2_ coating of N_ALD_ = 150 at 300°C, which did not undergo any visible changes (i.e., no needles were grown). This is in good agreement with the XRD analysis in Figure 2B that this ALD TiO_2_ coating was crystalline. As anatase is thermodynamically stable, the anatase coating completely prevents reaction between water and the TiO_2_ nanotube wall at room temperature (Wang et al., 2011).

Other works on water treated TiO_2_ nanotube layers suggested that the formation of nanoparticles on the tube walls is closely related to the growth of anatase crystals, due to the structural rearrangement of TiO${}_{6}^{2-}$ octahedral induced by water (Liao et al., 2011; Wang et al., 2011; Cao et al., 2016). Under extreme conditions (extensive soaking periods) it may eventually result in the transformation of hollow nanotubes to solid nanowires, or completely collapsed nanotube walls. Interestingly, the solid-state growth and dissolution-precipitation mechanism were proposed based on Yanagisawa and Ovenstone's model (Yanagisawa and Ovenstone, 1999), but it took more than 10 years to reveal this effect also for the amorphous anodic TiO_2_ nanotube layers (Liao et al., 2011; Wang et al., 2011).

Somewhat surprisingly, in the present work, the morphological changes of blank nanotube layers in Figure 3 are not accompanied by structural modification (amorphous to anatase) as of those reported in the literature. To understand the present phenomena, it is helpful to revisit the formation mechanism of the anodic TiO_2_ nanotube layer in fluoride-containing electrolytes (Macak et al., 2007; Lee et al., 2014). Briefly, the presence of F^−^ ions enables the formation of the fluoride-complex [TiF_6_]^2−^ ions, and the formation of tubular TiO_2_ is a competition between the solvatization of Ti^4+^ to [TiF_6_]^2−^ and the oxide formation. However, under the absence of an electric field (driven by an applied voltage), at the oxide/water interface, the reaction turns to a self-induced oxide formation, which translates into the nucleation of needle-like nanoparticles observed in Figure 3 (7 days). Apparently, the dissolution-precipitation mechanism is now inclined to the precipitation process, and the continuous precipitation leads to the copious quantity of nanoparticles as seen in Figure 3 (28 days). Dissimilar to Wang et al. (2011) and Cao et al. (2016) where the dissolution process gradually dissolved the tube walls, in Figure 3 (28 days), distinguished walls are identified even though the nanotubes are covered by the nanoparticles. This further affirms that the process is dominated by a surface precipitation process without structural modification.

**Figure 6:**
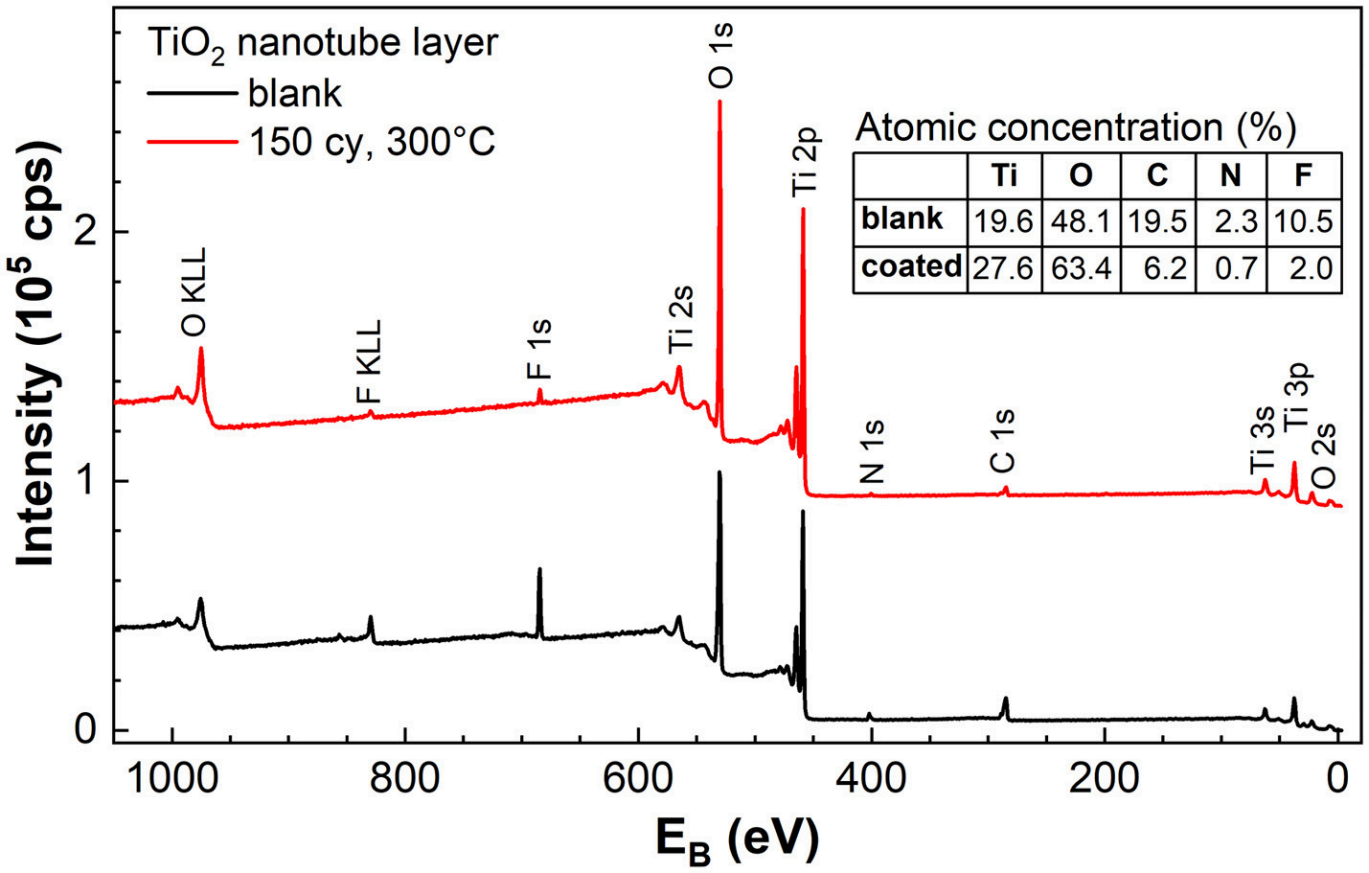
XPS survey spectra of blank amorphous and TiO_2_ coated (N_ALD_ = 150 at 300°C) TiO_2_ nanotube layers. The table in the inset shows the atomic concentration of the elements found in the survey spectra. Spectra were offset for better clarity.

Compositional analyses were carried out on the blank amorphous and TiO_2_ (N_ALD_ = 150 at 300°C) coated TiO_2_ nanotube layers by XPS. Besides Ti and O, the survey spectra in Figure 6 reveals the presence of F, C, and N in the nanotube layers. It has been pointed out that amorphous nanotube layers contain a considerable amount of F and C species from the anodization performed in the electrolyte consists of ethylene glycol and NH_4_F, specifically for double-walled nanotube layers (Albu et al., 2008) that were used also in this work. In particular, the presence of F content is associated with the high-field migration of electrolyte anions and the competition between small F^−^ ions and O^2−^ ions migration. For the successful formation of nanotubes, the inwards migration rate of F^−^ is twice to that of O^2−^, therefore accumulating a fluoride-rich inner layer especially toward the bottom of the tubes (Habazaki et al., 2007; Albu et al., 2008). In addition, an ultrathin fluoride-rich layer is also present at the outer walls, i.e., between individual tube walls, caused by the plastic-flow mechanism which pushes the nanotubes upward from the bottom of nanotubes/Ti interface during the tubes formation, hence promotes the F^−^ along the tube walls (Berger et al., 2011). The fluoride-rich, double-walled morphology is well-documented with the support by EDX, XPS, High Resolution Transmission Electron Microscope (HR-TEM), High Angle Annular Dark Field Scanning TEM (HAADF-STEM) Auger Electron Spectroscopy (AES) and Time-of-Flight Secondary-ion Mass Spectrometry (ToF SIMS) depth-profiling measurements (Albu et al., 2008; Berger et al., 2011; So et al., 2017; Dronov et al., 2018).

As a result, the as-anodized nanotube layers are usually subjected to thermal annealing at elevated temperatures for crystallization as well as for the removal of the F and C species (Albu et al., 2008). A clear double-walled morphology is visible after appropriate annealing process due to the removal of C in the form of CO_2_ that leads to the separation of the inner and outer walls, which have been shown in our previous works (Sopha et al., 2017b; Motola et al., 2018) and in other reports (Albu et al., 2008; So et al., 2017; Mohajernia et al., 2018). Without the annealing process, a substantial amount of C is noted in the amorphous nanotube layer as shown in Figure 6. The inner and outer walls remain intact and thus, the double-wall effect cannot be visualized in the images in Figure 1.

Comparing the blank and coated nanotube layers, the breakdown of the atomic concentration of each element tabulated in the inset of Figure 6 shows that the amount of F, C, and N is significantly reduced as a result of the ALD TiO_2_ coating. The C contamination can be partially assigned to the adventitious C resulting from the exposure to the air ambient. Whereas, the presence of F and N in the TiO_2_ coating is related to the diffusion of these two elements from the nanotube walls to the coating during the ALD process at elevated temperature (performed at 300°C). Nevertheless, the traces of F (2.0%) and N (0.7%) are almost negligible. Therefore, without sufficient F^−^ ions on the surface of anatase TiO_2_ coating (N_ALD_ = 150 at 300°C) at the water/coating interface, water annealing effect was not observed even after prolonged soakings. The same conclusion was reached by Cao et al. (2016) where the presence of a higher amount of F^−^ ions accelerated the growth of TiO_2_ nanoparticles.

When an amorphous TiO_2_ coating was added to the nanotube layer, forming nanotube/coating/water configuration, the additional coatings prepared by ALD (without F^−^ ions) served as a protective layer (similar function as the anatase coating discussed above) to separate the tube walls and water. Due to the great adhesion of the coatings to the nanotubes, there is no direct contact between F^−^ ions ([TiF_6_]^2−^ ions) and water. Hence, we observed in Figure 4 that nanoparticles were not formed on the tube walls up to 14 days of soaking. However, at the nanotube/coating interface, the F^−^ ions gradually attack both sides of TiO_2_ and the thinner coatings are more prone to F^−^ ions transport across the entire coating, as the F^−^ ions are very small and mobile. The thinner coatings may be eventually consumed by the F^−^ ions, and the tube walls may be partially exposed to the water which may result in higher precipitation and growth of more nanoparticles. As the self-induced precipitation process occurs in a slow manner, an extended duration is required to observe the soaking effect. After 28 days of soaking (Figure 5), the most prominent effect (highest amount of nanoparticles) is credited to the thinnest coatings of N_ALD_ = 10, followed by N_ALD_ = 50. For these two coating thicknesses, the deposition temperatures did not have a significant effect. However, for N_ALD_ = 150, the amorphous coating deposited at 225°C has fewer nanoparticles than that at 150°C.

**Figure 7:**
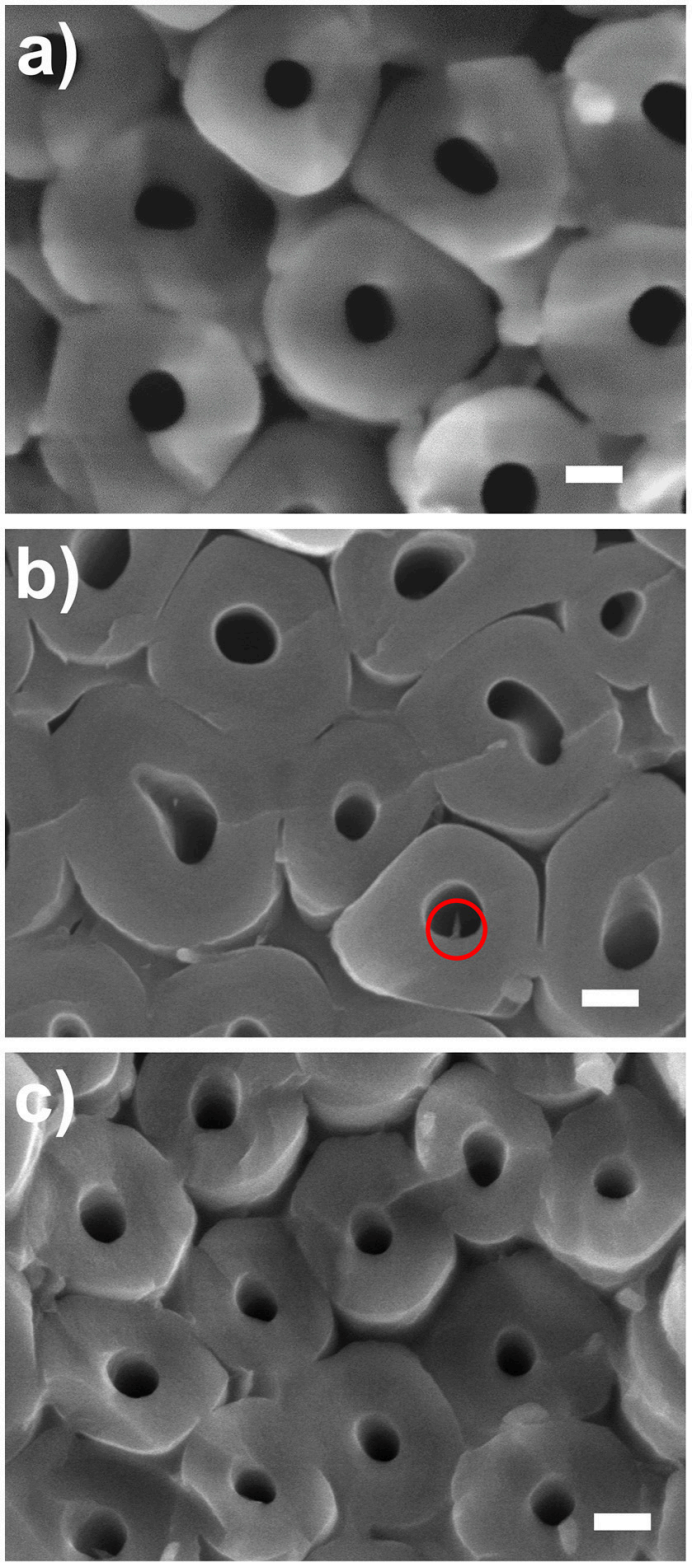
SEM images of bottom parts of the TiO_2_ nanotube layers taken close to the Ti substrate. **(a)** N_ALD_ = 10 at 150°C soaked for 14 days, N_ALD_ = 150 at **(b)** 150°C and **(c)** 225°C soaked for 28 days. All scale bars are 100 nm. The circle in **(b)** shows a needle found in an inner tube wall.

Further inspection of the bottoms of the TiO_2_ coated nanotube layers was carried out. At first, we inspected TiO_2_ coated (N_ALD_ = 10 at 150°C TiO_2_) nanotube layer, which is the lowest coating thickness deposited at the lowest temperature applied in this work, as a representative one for all coated TiO_2_ nanotube layers soaked for 14 days. The corresponding SEM image is shown in Figure 7a which confirms that no needles were grown for soakings up to 14 days. Note that the images shown in Figure 7 are representative image based on the extensive analyses on a broad range of nanotube samples produced by the corresponding conditions.

For further verification, we compared the bottoms of the TiO_2_ coated nanotube layers for N_ALD_ = 150 at 150° and 225°C and reached the same conclusion. Limited needles were discovered for 150°C in Figure 7b and almost no needle was detected for 225°C in Figure 7c. This is ascribed to the different densities of the TiO_2_ coating during the ALD process as higher deposition temperature generally promotes the interconnection between the grains (Aarik et al., 1995; Saha et al., 2014). Thus, for identical thicknesses of N_ALD_ = 150, the film density is higher at 225°C and it can better resist the attack of F^−^ ions.

Similar morphology of TiO_2_ nanoparticles coated TiO_2_ nanotube layer in Figure 3 (28 days) is observed for the renowned “TiCl_4_ treatment” often carried out to decorate the TiO_2_ nanotube layer by additional TiO_2_ nanoparticles for DSSCs (Meen et al., 2012; So et al., 2015). Likewise, the as-deposited TiO_2_ nanoparticles produced via hydrolysis of TiCl_4_ are amorphous and conventional thermal annealing is required to crystallize the nanoparticles. A major difference between TiCl_4_ treatment and water soaking is that the growth rate of TiO_2_ nanoparticles is much slower in the present case. We have presented that the nanotube layer was completely decorated by nanoparticles after 30 min of treatment in a TiCl_4_ bath (Sopha et al., 2017b), in line with other works (Meen et al., 2012; So et al., 2015). This is ascribed to the very reactive TiCl_4_ precursor and considerably high reaction (chemical bath) temperature at 70°C, which accelerate the growth process. As for water soaking, the precipitation is a self-induced process and much longer duration is required to accumulate a comparable quantity of nanoparticle deposits. It has also been confirmed that higher soaking temperature and longer reaction time promote the growth rate of TiO_2_ nanoparticles in a water bath (Krengvirat et al., 2013; Cao et al., 2016).

Altogether, these results indicate that the thin TiO_2_ coatings act as a protective layer to maintain the smooth tubular morphology of the as-anodized nanotube layers in the amorphous state for more than 14 days, while the unprotected nanotube layers hardly sustain 7 days of soaking. This generously increases more than twice of the initial lifespan of the smooth amorphous TiO_2_ nanotube layers which offers a stable platform for cell culturing and drug delivery testing typically carried out in the time scale from 3 to 20 days (Peng et al., 2009; Hu et al., 2012; Feng et al., 2016; Kaur et al., 2016). Moreover, it should be emphasized that a smooth morphology is usually favored for cell culturing, as the cell spreading and the cell survival rate is influenced by the morphology of TiO_2_ supporting layer (Park et al., 2007; Peng et al., 2009; Tian et al., 2015). In addition to the water soaking experiments, we also performed soakings in PBS with identical conditions (temperature, duration) for all blank and ALD TiO_2_ coated TiO_2_ nanotube layers. All these nanotube layers have revealed extreme stability in PBS. As shown in Supplementary Figures 1 and 2, no structural and morphological changes were observed even after 28 days due to the disruption of the precipitation kinetics by the inorganic species in the buffer solution. Although the full mechanism is not well-understood yet, this observation is in accord with Cao et al. (2016).

Overall, we recommend performing 150 ALD cycles of TiO_2_ coating, equivalent to 8.4 nm thicknesses at 225°C which is sufficiently thick for effective protection for the nanotube layers whilst keeping the amorphous state. Among all the amorphous coatings after 28 days of soaking, this condition has the fewest nanoparticles on the nanotube walls, evidenced in Figures 5 and 7.

## Conclusion

We proposed the utilization of thin ALD TiO_2_ coatings to protect 1D TiO_2_ nanotube layers against morphological changes within prolonged water soaking experiments. Thin and conformal TiO_2_ coating of N_ALD_ = 10, 50, and 150 corresponding to 0.56, 2.8, and 8.4 nm in thickness, respectively, were deposited by ALD at temperatures 150°, 225°, and 300°C within 5 μm amorphous TiO_2_ nanotube layers, which yielded amorphous and anatase coatings. The uncoated nanotube layers underwent significant morphological changes with additional nanoparticles formed on the nanotube walls after extensive soakings up to 28 days. The formation of the nanoparticles was related to the reaction between residual F^−^ ions (present in the nanotube walls) and water in a self-induced precipitation mechanism. The additional TiO_2_ coatings delayed the soaking effect and preserved the nanotube walls for a minimum of 14 days. Overall, the optimum coating was credited to N_ALD_ = 150 (8.4 nm) deposited at 225°C. The combination of identical materials by different preparation techniques sustains the amorphous state and tubular morphology of 1D TiO_2_ nanotube layers for biomedical applications as an example.

## Author Contributions

SN carried out the soakings and wrote the manuscript. HS synthesized the nanotubes and helped with the soakings. RZ and JP deposited the coatings. LH and VB carried out the SEM measurements. ZS measured and analyzed the XRD results. FD measured and evaluated the XPS results. JM designed the experiments, advised the results, corrected the manuscript and obtained the funding. All authors discussed, read, and approved the manuscript.

### Conflict of Interest Statement

The authors declare that the research was conducted in the absence of any commercial or financial relationships that could be construed as a potential conflict of interest.
