# Supplementary material for: TiO2 ALD Coating of Amorphous TiO2 Nanotube Layers: Inhibition of the Structural and Morphological Changes Due to Water Annealing
Source: Front Chem. 2019 Feb 1;7:38. doi: 10.3389/fchem.2019.00038 (PMC6367259; doi:10.3389/fchem.2019.00038)
Supplement: Supplementary file 1 [file Data_Sheet_1.docx]

Supplementary Material


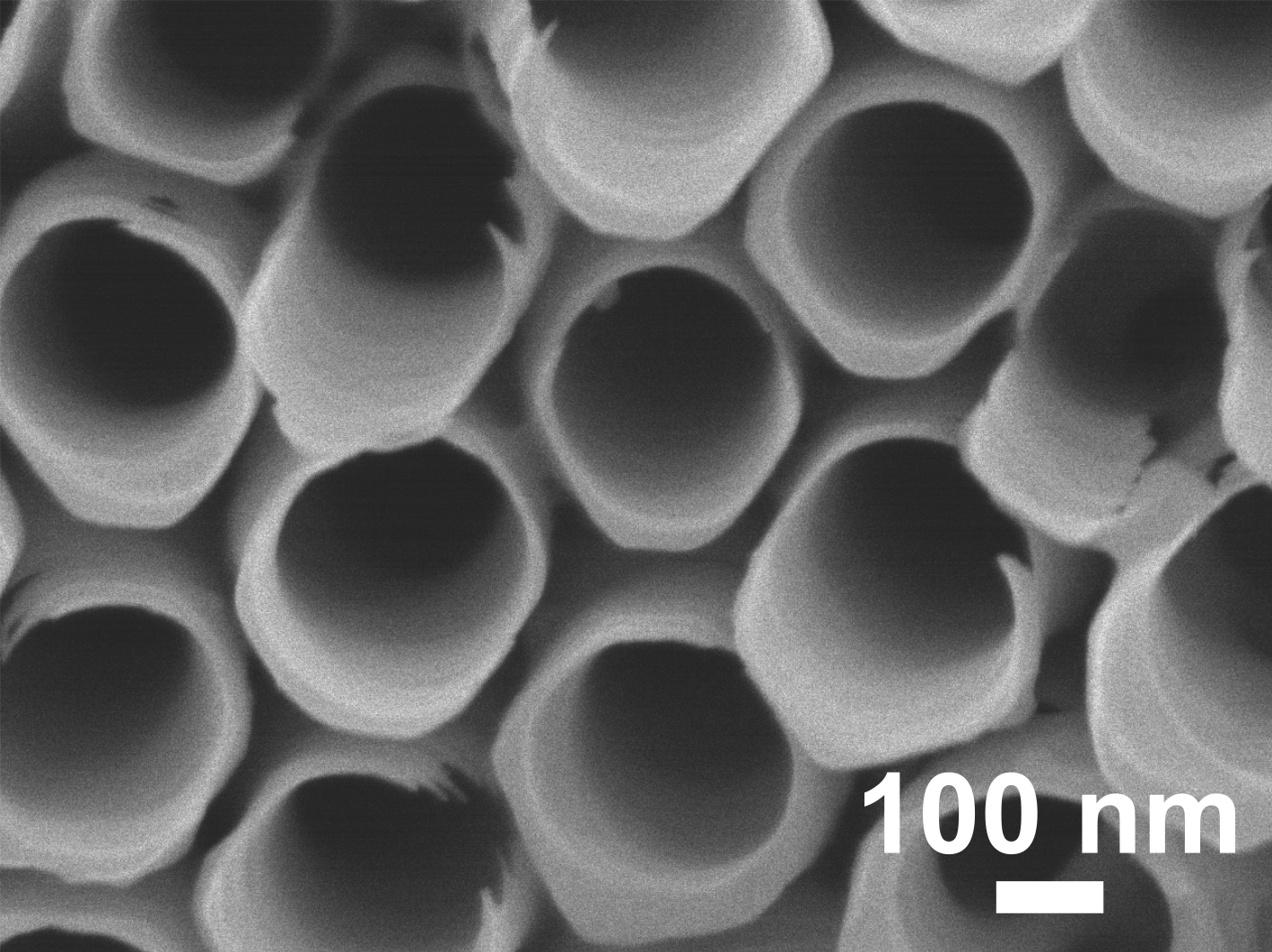


Supplementary Figure 1. SEM images of blank TiO_2_ nanotube layers soaked in phosphate buffer solution (PBS) for 28 days.


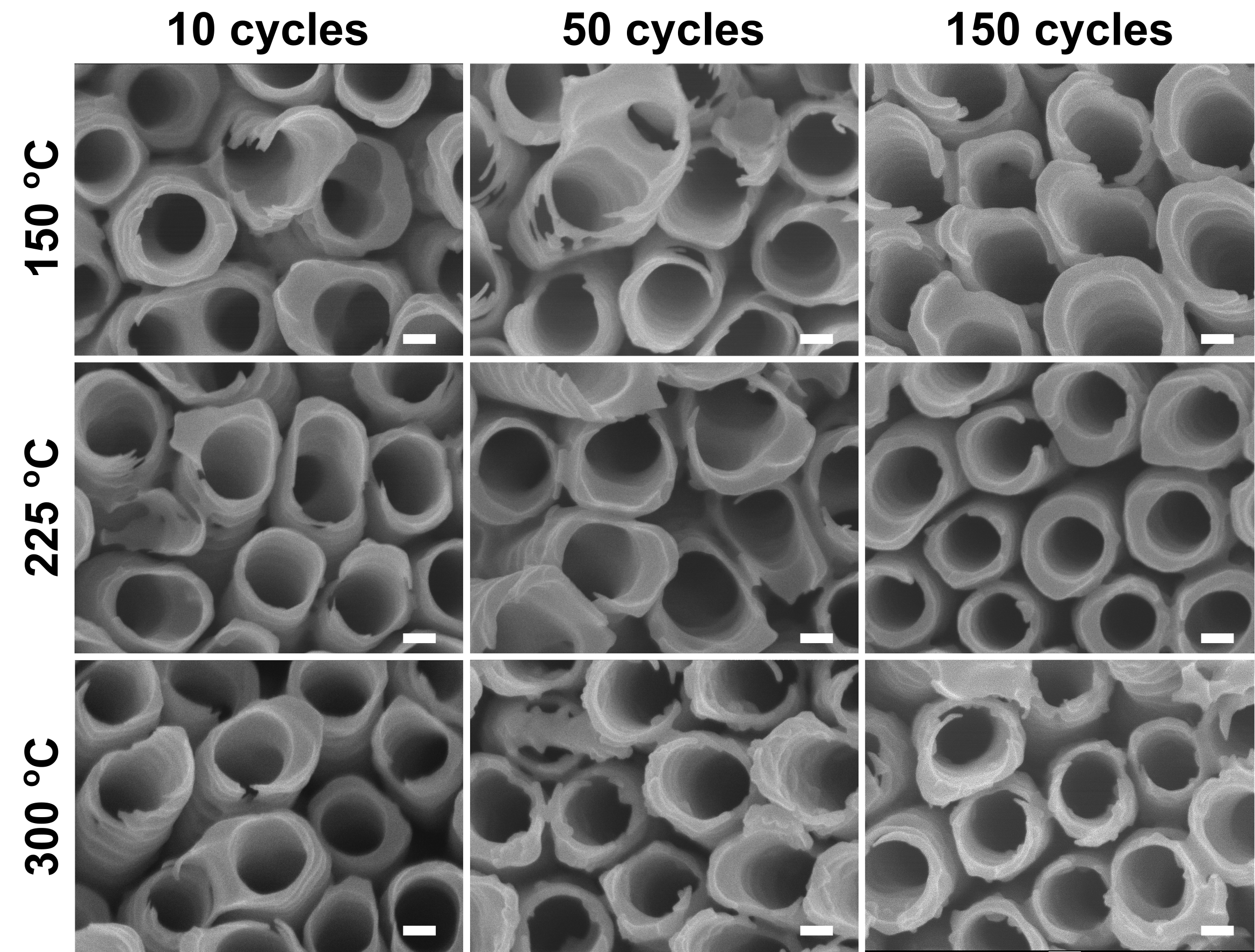


**Supplementary Figure 2.** SEM images of ALD TiO_2_ coated TiO_2_ nanotube layers soaked in PBS for 28 days. All scale bars are 100 nm.
